# ﻿*Elachistadimicatella* sensu auctt.—a complex of neglected species diversity (Lepidoptera, Elachistidae) from European mountain systems

**DOI:** 10.3897/zookeys.1212.126598

**Published:** 2024-09-16

**Authors:** Lauri Kaila, Peter Huemer

**Affiliations:** 1 Finnish Museum of Natural History, Zoology Unit, University of Helsinki, P.O.Box 17, FI-00014, Helsinki, Finland University of Helsinki Helsinki Finland; 2 Tiroler Landesmuseen Betriebsges.m.b.H., Natural History Collections, Krajnc-Str. 1, A-6060 Hall in Tirol, Austria Tiroler Landesmuseen Betriebsges.m.b.H., Natural History Collections Hall in Tirol Austria

**Keywords:** Cryptic diversity, DNA barcoding, Elachistinae, endemic, montane, morphology systematics

## Abstract

*Elachistadimicatella* Rebel, 1903, has so far been considered a species in Europe with restricted distribution from Ukraine to western France. The species occurs on mountainous regions. However, the in-depth analysis of a taxonomically uncertain species of *Elachista* from the Cottian Alps (Italy), especially through DNA barcoding and subsequent morphological studies, led to the realization that individuals previously identified as *E.dimicatella* from the Cottian Alps and the Pyrenees were misidentified. According to our research, a total of three species can be differentiated: *E.dimicatella* from Carpathians and its former junior synonym *E.niphadophanes* Meyrick, 1937, **sp. rev.**, from the Pyrenees, as well as the newly described *E.cottiella***sp. nov.** from southwestern Alps, hitherto incorrectly identified as *E.dimicatella*. Diagnostic features of the three species are discussed and illustrated. *Elachistadimicatella* and *E.niphadophanes* are redescribed.

## ﻿Introduction

Elachistinae is an extremely diverse subfamily of micromoths with more than 800 valid species worldwide placed in 10 genera (Kaila 2019). Furthermore, many undescribed species are known, and further taxa are expected to be discovered from large and virtually unexplored areas, particularly in Asia, Africa, and South America. Conversely, the European fauna is comparatively well explored, although new species are described almost every year, particularly from the Mediterranean. Additionally, the larger mountain systems of Europe, such as the Alps or the Dinarides, are likely to harbor a considerable potential for previously unrecognized species diversity. Therefore, it was not a big surprise that, as part of a comprehensive molecular survey of the regional Lepidoptera fauna in the northern Cottian Alps, several not identifiable species, including an *Elachista* sp., were discovered (Huemer and Wieser 2023). Morphology and DNA barcodes grouped this taxon as part of the diverse but taxonomically insufficiently revised *Elachistabifasciella* group first defined by Traugott-Olsen and Nielsen (1977), which, according to DNA barcoding with an extensive taxon sampling by Mutanen et al. (2013), encompasses a surprisingly large amount of cryptic diversity. However, DNA barcodes of the southwestern alpine taxon were previously unknown. They grouped as the nearest neighbour to *E.dimicatella* Rebel from the Ukrainian Carpathians, but with a significant genetic distance of about 6%. Subsequent research demonstrated that specimens previously identified as *E.dimicatella* from the southwestern Alps (Parenti and Varalda 1994) actually belong to the beforementioned morphologically and genetically distinct species, apparently without an available name. Investigations of type material of another taxon synonymized with *E.dimicatella* from the French Pyrenees, *E.niphadophanes* Meyrick, finally confirmed a third species within this hitherto overlooked species group.

## ﻿Materials and methods

The present paper is based on material from the following collections:

**MZH** Finnish Museum of Natural History, Helsinki, Finland

**MNHN** Museé National d´Historie Naturelle, Paris, France

**NHMW** Naturhistorisches Museum, Vienna, Austria

**TLMF** Tiroler Landesmuseum Ferdinandeum, Innsbruck, Austria

**ZSM** SNSB-Zoologische Staatssammlung München, Munich, Germany

Material of the new species collected by PH was attracted to artificial light (UV tubes) or bred (Parenti and Varalda 1994).

### ﻿Dissection

The terminology of genitalia follows the standard work of Traugott-Olsen and Nielsen (1977), with some modifications by Kaila (1999, 2011). To stain the male genitalia, an aqueous solution of red (yellow) eosin was used. The abdominal pelt of male specimens, and both the abdominal pelt and the genitalia of female specimens, were gently stained using chlorazol black.

### ﻿DNA barcodes

Tissue samples (dried legs) of three specimens of the new species were prepared for DNA barcoding (Hebert et al. (2003) processed at the Canadian Centre for DNA Barcoding (CCDB, Biodiversity Institute of Ontario, University of Guelph), following deWaard et al. (2008). In addition, 76 public and private barcode sequences of nine species of *Elachista*, considered to belong to the *Elachistabifasciella* species group, in the Barcode of Life Data Systems (BOLD; Ratnasingham and Hebert 2007; Ratnasingham 2018) were used for analysis. All barcodes, except for a single shorter sequence of *E.dimicatella*, range between 550 and 658 bp. Further details of sequenced material including complete voucher data and images of specimens can be accessed in the public dataset DS-ELACDIMI “Elachistadimicatella species group” https://doi.org/10.5883/DS-ELACDIMI in the Barcode of Life Data Systems (BOLD) (Ratnasingham and Hebert 2007; Ratnasingham 2018). Sequences from the dataset were finally submitted to GenBank. Degrees of intra- and interspecific variation of DNA barcode fragments were calculated using the Kimura two-parameter model on the platform of BOLD systems v. 4.0. (https://boldsystems.org). Calculation of intraspecific distance was furthermore normalized with BOLD calculation tools to reduce bias in sampling at the species level.

Sequences were assigned to Barcode Index Numbers (BIN). BINs were automatically calculated for records in BOLD that comply with the DNA Barcode standard (Ratnasingham and Hebert 2013).

A neighbour-joining tree was constructed using the Kimura-2-parameter model in MEGA7 (Kumar et al. 2016).

### ﻿Photographic documentation

Photographs of adults were taken with an Olympus OM-D Mark III camera and a 60 mm macro lens. Genitalia photographs of *E.niphadophanes* with a Zeiss Axiolab 5 microscope, mounted with an Olympus OM-D Mark III camera; 60 to 90 stacked photographs were edited using Helicon Focus 4.8 and Adobe Photoshop 6.0. Other genitalia images were taken with a Leica DM4000 B LED camera, and were edited, and the plates of each specimen assembled using several versions of Corel Photo-Paint included in CorelDRAW Graphics suite. Comparison of the length of the phallus in relation to the valva was measured as the longest line from the base of the sacculus to the apex of the cucullus. In the assembled male genital plates the true length of the phallus as compared with other parts of the genitalia is shown with the phallus positioned in the left side of the genitalia. The magnified images of the phallus and the juxta do not reflect their relative sizes among the species. The same applies to the “whole genitalia with phallus” part. These images are not at the same scale between images. The size of the genitalia correlates with the size of the specimen, so the wingspan can be used as a proxy for the relative sizes of the genitalia.

### ﻿Distribution map

The background (Hillshade: transparency 27.4%) was integrated using WMS (Web Map Service) from the address osm-wms.de (license: CC BY-SA), whose original data is based on satellite data (https://srtm.csi.cgiar.org/). The administrative boundaries were taken from the European site https://ec.europa.eu/eurostat/web/gisco/geodata/administrative-units/countries (license: CC BY 4.0). The water network is from https://land.copernicus.eu/ (digital rights: full, open and free access).

## ﻿Results of molecular analysis

**Figure 1.**
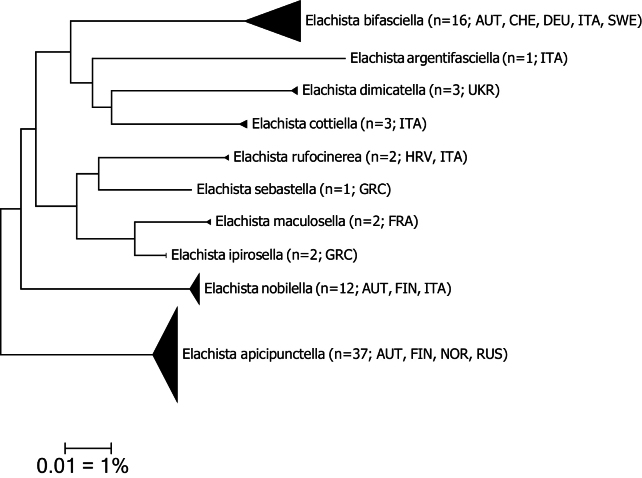
Neighbour-joining tree of *Elachista* species (Kimura-2-parameter, built with MEGA7 cf. Kumar et al. 2016). Note: the scale bar only applies to internal branches between species. Width of triangles represent sample size, depth the genetic variation within the cluster. Source: DNA Barcode data from BOLD, DS-ELACDIMI (Barcode of Life Database, cf. Ratnasingham 2018).

Molecular analysis is based on 79 DNA barcodes sequences for nine *Elachista* species belonging to the *Elachistabifasciella* species group. The interspecific distances to the nearest neighbour in this dataset vary from 4.14% to 8.59% per species pair. Intraspecific barcode variation is distinctly lower but based on only limited material for few species. It exceeds 2% only in *E.bifasciella* which clusters in 2 BINs, one geographically limited to southern Austria, whereas all other specimens cluster in a single and unique BIN (Table 1, Fig. 1).

**Table 1.** Intraspecific mean K2P (Kimura-2-Parameter) divergences, maximum pairwise distances, Barcode Index Number (BIN), nearest species, distance to nearest neighbor (NN) (distances in %) of *Elachista* spp. Source: DNA Barcode data from BOLD, DS-ELACDIMI (Barcode of Life Database, cf. Ratnasingham 2018).

Species	Mean intra	Max intra	BIN	Nearest species	Distance to NN
*Elachistaapicipunctella*	0.12	1.24	BOLD:AAE0019	*Elachistaipirosella*	6.65
*Elachistaargentifasciella*	N/A	0	BOLD:ACT4739	*Elachistacottiella*	8.59
*Elachistabifasciella*	0.75	2.89	BOLD:AAK9442 BOLD:AEC6228	*Elachistadimicatella*	8.36
*Elachistacottiella*	0.31	0.46	BOLD:AEM6237	*Elachistadimicatella*	6.57
*Elachistadimicatella*	0.21	0.24	BOLD:AAV6449	*Elachistacottiella*	6.57
*Elachistaipirosella*	0	0	BOLD:ABA2345	*Elachistamaculosella*	2.18
*Elachistamaculosella*	0.15	0.15	BOLD:ABW4899	*Elachistaipirosella*	2.18
*Elachistanobilella*	0.13	0.32	BOLD:AAF6140	*Elachistadimicatella*	6.75
*Elachistarufocinerea*	0.15	0.15	BOLD:ABV9577	*Elachistasebastella*	4.75
*Elachistasebastella*	N/A	0	BOLD:ACE1105	*Elachistaipirosella*	4.14

### ﻿Taxonomy

#### ﻿ Elachista dimicatella

Taxon classificationAnimaliaLepidopteraElachistidae

Rebel, 1903

0C1F91EA-AD5E-5025-8F2B-ECC26239C098

Elachista dimicatella Rebel, 1903: 100. Type locality: Ukraine, Marmaros.

##### Material examined.

***Lectotype*** ♂, designated by Gaedike (1975: 244) (NHMW). [pictures of adult and genitalia examined]

**Poland** • 1♂; Tatra Mts., Kościeliska Valley; 1000 m a.s.l.; 49.30°N, 19.93°E; 22 Jun 1987; ex larva from *Sesleriatatrae*; J. Buszko leg.; L. Kaila prep. 587; *Elachistadimicatella* Hering, L. Kaila det. 2004; 1♀, same collecting data but host plant *Alopecuruspratensis*; MZH • 1♂; Tatra Mts., Wąwóz Kraków, DV15, 1000 m a.s.l.; 49.23°N, 19.86°E; 2 Jun 2020; T. Baran leg.; gen. prep. J. Tabell 4390; DNA barcode voucher 16240 Lepid. Phyl.; MZH • 1♂; Tatra N. P., Mt. Bobrowiec; 1300 m a.s.l.; 49.24°N, 19.78°E, spruce forest; 16 Jun 1996; K. Mikkola leg.; MZH • 2♂; Tatra N. P., Jesiolów Zl; 1400 m a.s.l.; 27 Jul 1997; K. Mikkola leg.; MZH • 1♂; Tatra N. P., Chochołowska Valley, Bobroviec; 1250–1350 m a.s.l.; 49.16°N, 19.83°E; 24 Jul 1997; K. Mikkola leg.; L. Kaila prep. 6345; MZH • 1♂; Tatra Mts., Giewont, Mnich Malolacki; 1500 m a.s.l.; 49.25°N, 19.93°E; 24 Jul 1997; K. Nupponen & J. Junnilainen leg.; MZH • 3♀; Tatra Mts., Kominiarski Wierch; 1700–1800 m a.s.l.; 49.24°N 19.83°E; 28 Jul 1997; K. Nupponen & J. Junnilainen leg.; L. Kaila prep. 6378; MZH • Tatra Mts.;1400 m a.s.l.; 25.–28 Jun. 1977; ex larva from *Sesleriacoerulea*, *Poa*; H. Steuer leg; ZSM.

**Ukraine** • 6♂ 2♀; Ivano-Frankivsk oblast, Verkhovyna district, Mt. Chivchen; 1600–1760 m a.s.l.; 48.15°N, 24.82°E; J. Kullberg & T. Lievonen leg.; L. Kaila prep. 4653, 4827, 6383; DNA barcode vouchers 16160, 16161 Lepid. Phyl.; MZH • 6♂ 2♀; Ivano-Frankivsk oblast, Verkhovyna district, Burkut region; 47.908°N, 24.698°E; 3.–5 Jun 2003; J. Kullberg & T. Lievonen leg.; L. Kaila prep. 6379; DNA barcode voucher 16159 Lepid. Phyl.; MZH.

##### Diagnosis.

*Elachistadimicatella* is externally characterized by the creamy white head, the white base of the nearly black forewing reminiscent of *E.diederichsiella* Hering, straight and relatively narrow median fascia, and the usually medially confluent subcostal (tornal) and costal spots, often forming a steep angle towards apex. The antennae of the male are unicolourous grey in the male, in the female paler in apical half; in *E.cottiella* the antennae of both sexes are unicolourously dark grey, in *E.niphadophanes* the antennae of the male vary. In the male genitalia *E.dimicatella* is close to *E.niphadophanes* from which it differs by the somewhat longer phallus. From *E.cottiella* it is distinguished by the white pattern of forewings, that being somewhat silvery in *E.cottiella*. Subcostal and tornal spots are clearly separate in *E.cottiella.* In the male genitalia the most distinctive difference between *E.dimicatella* and *E.cottiella* is the markedly longer phallus in *E.cottiella* in which the shape of the juxta lobes is more rounded than in the other species. The antrum is significantly longer and narrower in *E.cottiella* than in *E.dimicatella* in which it is distinctly convex. Apophyses anteriores are thinner in *E.dimicatella* than in *E.cottiella*. More detailed diagnosis between *E.dimicatella* and *E.cottiella* are presented in the diagnosis of the latter species. The female of *E.niphadophanes* is unknown.

##### Redescription.

**Figures 2–6.**
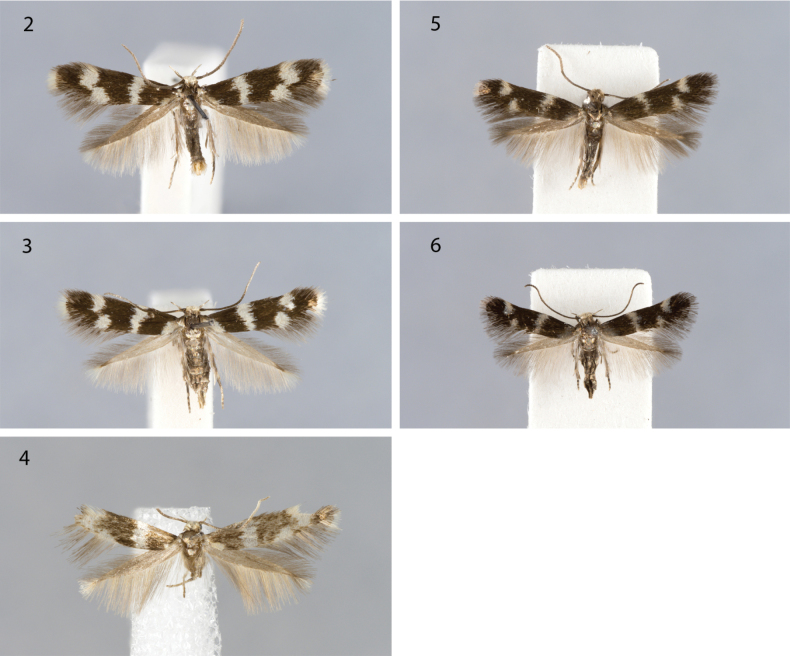
Adults. **2***Elachistadimicatella*, male, Poland **3***E.dimicatella*, female, Poland **4***E.niphadophanes*, male, France **5***E.cottiella* sp. nov., male, paratype, Italy **6***E.cottiella* sp. nov., female, paratype, Italy.

Habitus (Figs 2, 3). Wingspan 8.5–11 mm, male on average larger than female. Labial palpus ascending, approximately as long as diameter of head, off-white to silvery grey above, fuscous below; 3^rd^ segment often paler than 2^nd^ segment. Head creamy white; neck tuft varying from white or grey to almost black, thorax dark grey, tegula in basal half dark grey, white in distal half. Antenna entirely dark grey in male, distally a little paler in female. Fore- and midleg dark grey; hindleg outwards dark grey, inwards off-white with also spurs, tibia and tarsal articles distally off-white. Ground colour of forewing very dark brown to nearly black; base white especially on dorsal side; white transverse fascia of varying width at 1/3 forewing length; similarly coloured subcostal and tornal spot at 3/4 forewing length, confluent forming medially outward directed fascia, in female sometimes separate; fringe as ground colour, at termen white. Underside of fore- and hindwing dark grey with concolourous fringe.

**Figures 7–9.**
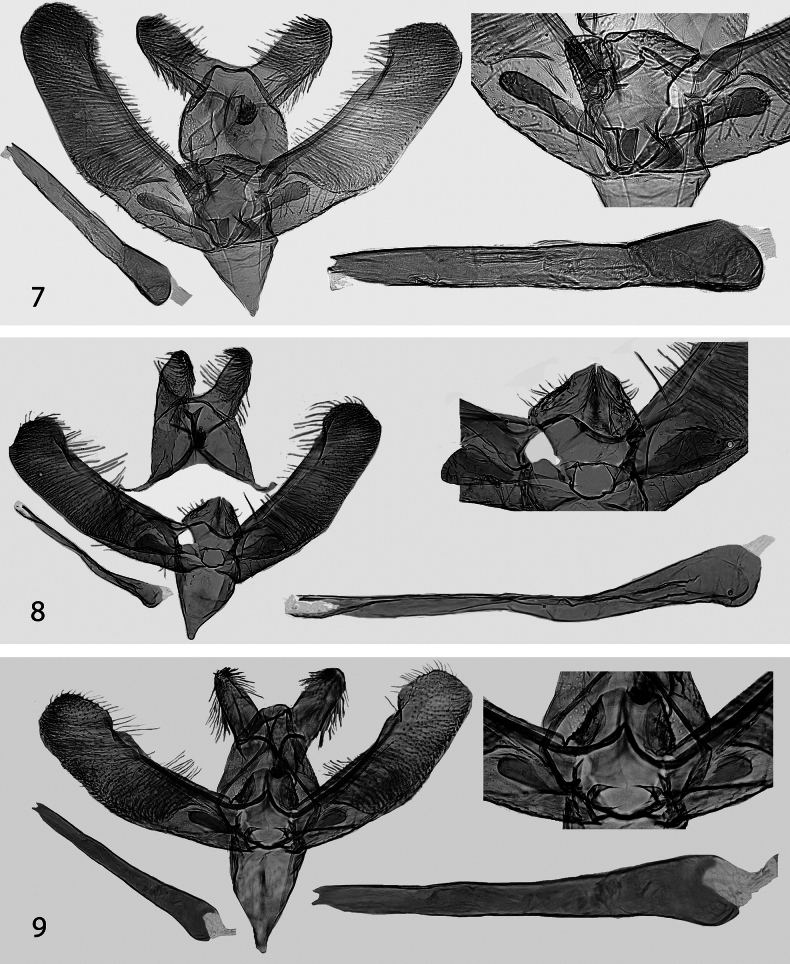
Male genitalia, with details enlarged **7***Elachistadimicatella*, Ukraine, slide 4827 L. Kaila **8***E.niphadophanes*, France, slide 7419 J. Nel **9***E.cottiella* sp. nov., paratype, Italy, slide 6347 L. Kaila.

Male genitalia (Fig. 7). Uncus lobes widely apart from each other, separated by convex posterior margin of tegumen, ventrolaterally directed, tongue-shaped, 2 × as long as wide, distally round. Spinose knob of gnathos small as compared to average size within the *E.bifasciella* group. Valva slightly bent, broadest medially, 3.6 × as long as wide at its widest part medially; basal fold of costa extended to distal 3/4 of valva where it meets distal fold forming indistinct hump. Digitate process ¼ as long as valva, narrow and parallel-sided, with a few setae distally. Mesial margin of juxta lobes straight, meeting distal margin at a right angle, distal margin nearly straight, broadly setose. Vinculum tapered, distally v-shaped, no distinct median ridge. Phallus 0.75 × as long as valva, straight, distal end bifurcate; without cornuti; caecum short, round, posterior opening dorsally projected.

**Figures 10, 11.**
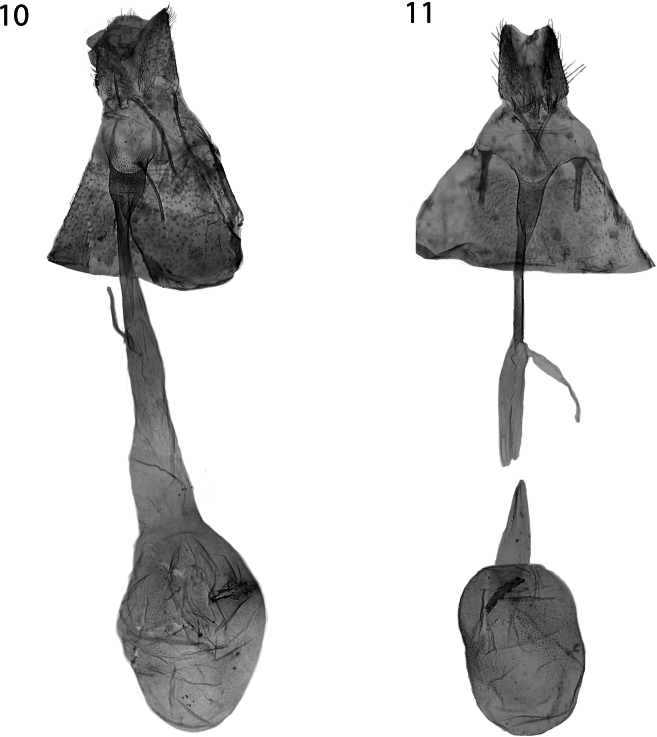
Female genitalia **10***Elachistadimicatella*, Ukraine, slide 6383 L. Kaila **11***E.cottiella* sp. nov., paratype, Italy, slide 6381 L. Kaila.

Female genitalia (Fig. 10). Papilla analis round in lateral view; membrane between papillae anales ventrally densely covered by minute spines, ventral margin otherwise with row of setae, setae longest at apex of papilla analis. Apophysis posterioris as long as papilla analis. Apophysis anterioris 2/3 × as long as apophysis posterioris. Ostium bursae in anterior margin of tergum 8, posterior margin convex; dorsal wall spinose; length of antrum slightly less than length of apophysis posterioris; antrum convex, colliculum posteriorly more and anteriorly less sclerotized, length as measured from anterior end of antrum to inception of ductus seminalis almost 3 × as long as apophysis posterioris; ductus bursae otherwise tubular, membranous, 1.5 × as long as antrum + colliculum, widened anteriorly, incepted in corpus bursae without clear limit; corpus bursae round, with small internal granules; signum elongate, dentate, broadest medially.

##### Molecular analysis.

BIN: BOLD:AAV6449 (*n* = 3). Intraspecific average *p*-distance within BIN is 0.15%, maximum distance is 0.15%. The nearest neighbour is *E.cottiella* (BIN: BOLD:AEM6237) at a distance of 6.41%.

##### Biology.

In the Polish Tatra Mountains the larvae were collected in late May and June, preferably feeding on *Sesleriatatrae* and *Deschampsiacaespitosa*, and furthermore on *Alopecuruspratensis*, *Calamagrostisarundinacea*, *C.villosa*, *Dactylisglomerata*, *Miliumeffusum*, and *Poaalpina*. Adults were observed from late June to July. The habitat is described as grassland on sunny slopes, exclusively on calcareous soil at altitudes between 1000 and 1800 m (Buszko and Baraniak 1989). Steuer (1976) gives a detailed description of the larva habits as leaf-miners in *Sesleriacoerulea*.

Several additional host-plants published by Parenti and Varalda (1994) cannot be attributed to a host-species and require confirmation.

##### Distribution.

Carpathians: Poland (mainly Tatra mts.) (Paluch et al. 2022), Slovakia (Laštůvka et al. 2018), Ukraine (type-locality; Ivano-Frankivsk region) (Fig. 13). A unique record from eastern Austria (Kasy 1980) requires confirmation. Occasional records from the Alps are considered as misidentifications, possibly representing the externally closely similar *E.argentifasciella* Höfner. *Elachistadimicatella* was originally described from Marmaros, which at that time was part of Hungary. However, after the collapse of the Austro-Hungarian monarchy, the type locality became part of what is now Ukrainian territory, a change that was mistakenly overlooked later (i.e., Gaedike 1975).

#### ﻿ Elachista niphadophanes

Taxon classificationAnimaliaLepidopteraElachistidae

Meyrick, 1937 sp. rev.

4C876C22-552F-5D26-B556-125048371D74

Elachista niphadophanes Meyrick, 1937: 100. Type locality: France, Forges d’Abel. Lectotype ♂, designated by Parenti (1972: 39) (MNHN). Synonymized by Steuer (1976: 174). [pictures of adult and genitalia examined].

##### Material examined.

**France** • 2♂; Pyrenees Orientales, Mosset, Col de Jau; 1450 m a.s.l.; 42.72°N, 2.25°E; 24 Jun.1998 [one without abdomen]; genitalia slide 7419JN; coll. Thierry Varenne • 1♂; Départment des Hautes-Pyrénees, Gripp, Col de Jau, Gripp; 42.96°N, 0.21°E; 8 Jul 1982; C. Gielis leg; MZH.

##### Diagnosis.

*Elachistaniphadophanes* is overall very similar to *E.dimicatella*, but the known specimens are somewhat smaller than males of *E.dimicatella*. They differ by the distally lighter colour of the antenna and the broader median fascia of the forewing in *E.niphadophanes*. From the similar *E.cottiella* it furthermore differs by several characters, particularly the colour of the antenna, white tipped tegulae, larger extension and white colour of forewing markings, and the white termen of the forewing. In the male genitalia the most distinctive difference between *E.niphadophanes* and *E.cottiella* is the much larger phallus in *E.cottiella*. The male genitalia are similar to those of *E.dimicatella*, but the phallus is somewhat smaller in *E.niphadophanes*.

##### Redescription.

Habitus [based on two worn male specimens and figure of lectotype] (Fig. 4). Wingspan 8–9 mm. Labial palpus ascending, approximately as long as diameter of head, off-white to silvery grey above, fuscous below; 3^rd^ segment purely off-white. Head white; neck tuft creamy white, thorax dark grey, tegula in basal half dark grey, white in distal half. Antenna dark grey-brown in basal 2/5, distal part can be paler grey. Mid- and hindlegs outwards grey, inwards off-white with also spurs, tibia and tarsal articles distally off-white. Ground colour of forewing dark brown; base white on dorsal side; broad white transverse fascia at 1/3 forewing length; similarly coloured subcostal and tornal spot at 3/4 forewing length, weakly confluent forming medially outward directed fascia; fringe as ground colour, at termen white. Underside of fore- and hindwing dark grey with concolourous fringe. Female unknown.

Male genitalia (Fig. 8). Uncus lobes apart from each other, separated by convex posterior margin of tegumen, ventrolaterally directed, tongue-shaped, 2 × as long as wide, distally round. Spinose knob of gnathos very small as compared to average size within the *E.bifasciella* group, round. Valva straight, slightly broadest in middle, basal fold of costa extended to distal 3/4 of valva where meeting distal lobe and forming distinct hump. Digitate process 1/4 × as long as valva, distally somewhat oblique with a few setae. Mesial margin of juxta lobes straight, meeting distal margin at a right angle, distal margin somewhat convex, laterally setose. Vinculum distally tapered into short saccus, no median ridge present. Phallus 0.6 × as long as valva, broadest in basal 1/4, straight, distal end bifurcated; without cornuti; caecum short, bulbous, posterior opening dorsally projected.

Female unknown.

##### Molecular analysis.

Unfortunately, no DNA barcode could be retrieved for this species.

##### Biology.

Host-plant and early stages are unknown. Host-plants from the Polish Tatra Mountains attributed to the former senior synonym *E.dimicatella* are not applicable for *E.niphadophanes*. Furthermore, host-plants published by Parenti and Varalda (1994) cannot be attributed to this species and likely most of them belong to the south-western alpine *E.cottiella*.

##### Distribution.

Pyrenees. With certainty only known from the type locality Forges d’Abel, from Col de Jau and Gripp (Pyrenees, France), from Cole de Jau published as *E.dimicatella* (Peslier et al. 2023) (Fig. 13).

##### Remarks.

*Elachistaniphadophanes* was described from two specimens (suggesting male and female sex) collected by Lhomme in the French Pyrenees (Forges d’Abel). Parenti (1972) dissected the only available syntype in MNHN and designated this specimen as lectotype. He furthermore mentioned three male specimens in coll. Lhomme. Later, Steuer (1976) incorrectly synonymized *E.niphadophanes* with *E.dimicatella* solely from an overall similarity of the male genitalia as figured by Parenti (1972) and based on written information from Parenti, that there would be no differences between the adults of the two taxa.

#### ﻿ Elachista cottiella sp. nov.

Taxon classificationAnimaliaLepidopteraElachistidae

70C4B1F3-85C4-5500-81D2-DADFFB9B4C04

https://zoobank.org/98E5AC18-54CE-4D00-B041-C46C4C8813B2

##### Type material.

***Holotype*. Italy** • ♂; Prov. Torino, Fenestrelle, Umg. Pracatinat, Forte delle Valli [type locality part of Orsiera-Rocciavrè Nature Park]; 45°2'17"N, 7°4'14"E; 1700–1720 m; 02 Jun 2022; P. Huemer leg.; DNA Barcode ID TLMF Lep 32861; TLMF.

**Figure 12.**
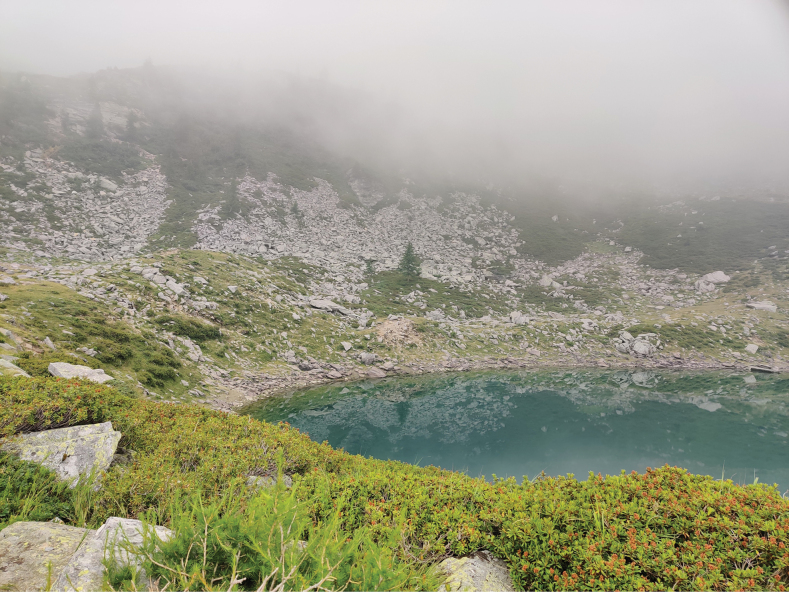
Habitat of *Elachistacottiella* sp. nov. (Italy, Torino, Lago Lauson).

**Figure 13.**
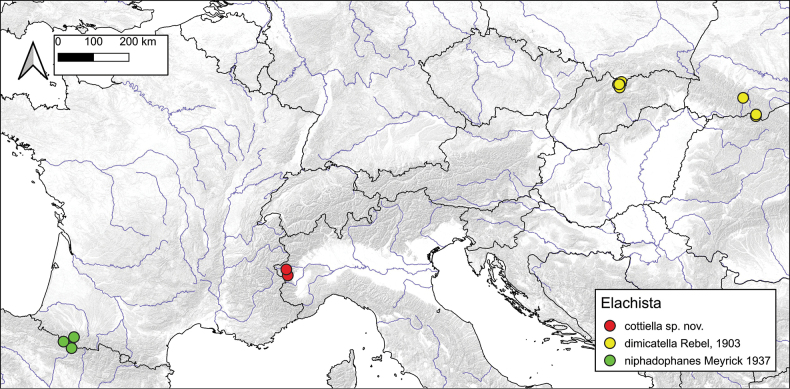
Proved records of *Elachistacottiella* sp. nov. (red dots), *E.dimicatella* (yellow dots) and *E.niphadophanes* (green dots).

***Paratypes*. Italy** • 7♂; prov. Torino, N.N. Conca Cialancia, Umg. Lago Lauson; 2030–2050 m; 44°53'16"N, 7°7'36"E; 17 Jul 2022; P. Huemer leg.; DNA Barcode IDs TLMF_Lep_33427, TLMF_Lep _33428; TLMF • 6♂ 4♀; Piemonte, Provonda → Ciai (Giaveno, to); 45°11'N, 7°28'E; reared from *Festucaovina* group; P. Varalda leg.; L. Kaila prep. 6346, 6347, 6380, 6381, J. Tabell prep. 4392, 4654; DNA Barcode ID samples 1641, 1632 Lepid. phyl. (unsuccessful); *Elachistadimicatella* det. U. Parenti; http//id.luomus.fi/GD1118-1127; MZH • 2♂ 2♀; Piemonte, Strada Giaveno, Provonda; 950 m; 25 + 28 May 1989; reared from *Avenellaflexuosa*; U. Parenti leg; ZSM.

##### Diagnosis.

*Elachistacottiella* differs from the closely related *E.dimicatella* and *E.niphadophanes* by the entirely grey antennae in both male and female, entirely grey tegulae, a narrower silvery-white medial fascia and smaller and separate subcostal and tornal spots of the forewing, and entirely dark fringe. In the former mentioned species, wing markings are broader with costal and tornal spots medially fused, and the tip of forewing fringe is white. The digitate process is shorter in *E.cottiella* and *E.niphadophanes* than in *E.dimicatella*. The lateral margins of the juxta lobes are more convex in *E.niphadophanes* than in *E.cottiella* and *E.dimicatella* thus making it more round than in the other species. The phallus of *E.niphadophanes* is shorter than in either other species, 0.6 × as long as valva, basally bulbous. The antrum is significantly longer and narrower in *E.cottiella* than in *E.dimicatella* in which it is distinctly convex. Apophyses anteriores are stouter in *E.cottiella* than in *E.dimicatella.* The female of *E.niphadophanes* is unknown.

##### Description.

Habitus (Figs 5, 6). Wingspan 6.5–8.5 mm (reared specimens), male on average larger than female. Labial palpus ascending, approximately as long as diameter of head, off-white to silvery grey above, broadly dark grey below. Head off-white to creamy, shiny; neck tuft varying from creamy to grey; thorax dark grey, tegula grey. Antenna entirely grey both in male and female. Legs shiny grey; hindleg inwards off-white with also spurs, tibia and tarsal articles distally off-white. Ground colour of forewing very dark brown to nearly black; base sometimes, in female in particular, silvery white on dorsal side; white or silvery, rather narrow transverse fascia of at 1/3 forewing length, metallic shine more pronounced in female; similarly coloured subcostal and tornal spot near apex of forewing separate, subcostal spot closer to apex than dorsal spot; fringe as ground colour. Underside of fore- and hindwing dark grey with concolourous fringe.

Male genitalia (Fig. 9). Uncus lobes widely apart from each other, separated by convex posterior margin of tegumen, ventrolaterally directed, tongue-shaped, 2 × as long as wide, distally round. Spinose knob of gnathos small as compared to average size within the *E.bifasciella* group, longer than wide. Valva straight or slightly bent, basal fold of costa extended to distal 3/4 of valva where meeting distal lobe and forming variably distinct hump. Digitate process 1/5 × as long as valva, parallel-sided, with a few setae distally. Mesial margin of juxta lobes slightly bent to median direction, meeting distal margin at a right angle, distal margin somewhat concave, laterally setose, lateral margin distinctly concave. Vinculum tapered, distally v-shaped, with indistinct median ridge. Phallus as long as valva, broadest in basal 1/4, straight, distal end bifurcate; without cornuti; caecum short, rounded, posterior opening dorsally projected.

Female genitalia (Fig. 11). Papilla analis round in lateral view; membrane between papillae anales ventrally densely covered by minute spines, evenly setose. Apophysis posterioris as long as papilla analis. Apophysis anterioris 2/3 as long as apophysis posterioris, stout. Ostium bursae in anterior margin of tergum 8, posterior margin convex; dorsal wall spinose; antrum elongate, hardly convex, longer than apophysis posterioris; length of colliculum as measured from anterior end of antrum to inception of ductus seminalis almost 2 × as long as apophysis posterioris; ductus bursae otherwise tubular, membranous, as long as antrum + colliculum, incepted in corpus bursae with clear limit; corpus bursae round, with small internal granules; signum elongate, dentate.

##### Molecular analysis.

BIN: BOLD:AEM6237 (*n* = 4). Intraspecific average *p*-distance within BIN is considerable with 1.1%, maximum distance is 2.09%. The nearest neighbour is BINBOLD:ACG7227 (*E.wieseriella* and an unspecified *Elachista* sp.) at a distance of 6.19%, and *E.dimicatella* (BIN: BOLD:AAV6449) with 6.41% distance.

##### Biology.

Host-plants insufficiently documented. All reared specimens available to us have been reared from *Avenellaflexuosa* and *Festuca* sp. in *rubra* group. Several host-plants from the Polish Tatra Mountains attributed to the former senior synonym *E.dimicatella* are not applicable for the new species. However, additional host-plants published by Parenti and Varalda (1994) could all belong to *E.cottiella*. *Elachistacottiella* was found on siliceous soil in montane to subalpine open grassland with adults attracted to UV lamps (Fig. 12).

##### Distribution.

Alps. With certainty only known from Piemont (Cottian Alps, Italy) (Fig. 13).

##### Etymology.

The new species name refers to the known distribution area.

## ﻿Discussion

The documentation of the diversity of European *Elachista* species has advanced considerably, not in the least due to the increasing implementation of molecular methods. The status of described species is still largely supported by the study of historical type material (Kaila 2019), but increasingly there are more publicly available DNA barcode data. Nevertheless, despite these advancements, the presence of undescribed species remains a significant challenge. The unexpected discovery of a previously overlooked or taxonomically misinterpreted species complex, as highlighted by Mutanen et al.’s (2013) extensive molecular investigations in this group, adds an element of surprise. It appears that the expanded geographical coverage within this family suggests the likelihood of further hidden diversity.

For instance, Huemer (2020) and Huemer and Wieser (2023) have identified additional cryptic species from the Alps lacking reference sequences in BOLD. The taxa now revised, and previously grouped under the name *E.dimicatella*, exhibit small but diagnostic differences in phenotype, genital morphology, and molecular characteristics (DNA barcode). Notably, all these species have been observed exclusively in allopatry, a pattern observed in many butterflies and moths across European mountainous regions (Fig. 13). The substantial variations in species compositions among genera of montane to alpine Lepidoptera in the Pyrenees, Alps, and Carpathians are well documented (Huemer and Tarmann 1992; Whitebread 2006; Huemer and Hausmann 2009; Huemer and Hebert 2011; Zlatkov and Huemer 2017).

## Supplementary Material

XML Treatment for Elachista dimicatella

XML Treatment for Elachista niphadophanes

XML Treatment for Elachista cottiella

## ﻿Additional information

### Author ORCIDs

Lauri Kaila https://orcid.org/0000-0003-0277-1872

Peter Huemer https://orcid.org/0000-0002-0630-545X

### Data availability

All of the data that support the findings of this study are available in the main text.
